# Safety and Efficacy of Selective Internal Radionuclide Therapy with ^90^Y Glass Microspheres in Patients with Progressive Hepatocellular Carcinoma after the Failure of Repeated Transarterial Chemoembolization

**DOI:** 10.3390/ph17010101

**Published:** 2024-01-11

**Authors:** Alexander Bellendorf, Nicolai Mader, Stefan P. Mueller, Samer Ezziddin, Andreas Bockisch, Hong Grafe, Jan Best, Juliane Goebel, Thorsten D. Pöppel, Amir Sabet

**Affiliations:** 1 Department of Nuclear Medicine, University of Duisburg-Essen, Hufelandstr. 55, 45147 Essen, Germany; alexander.bellendorf@ruhrradiologie.de (A.B.); stefan.mueller@uni-due.de (S.P.M.); andreas.bockisch@uk-essen.de (A.B.); hong.grafe@uk-essen.de (H.G.);; 2 MVZ Radiologie, Nuklearmedizin und Strahlentherapie Essen GmbH, Ruüttenscheider Str. 191, 45131 Essen, Germany; 3 Department of Nuclear Medicine, Clinic for Radiology and Nuclear Medicine, University Hospital, Goethe University Frankfurt, Theodor-Stern-Kai 7, 60590 Frankfurt am Main, Germany; n.mader@med.uni-frankfurt.de; 4 Department of Nuclear Medicine, Saarland University Medical Center, Kirrberger Straße, 66421 Homburg, Germany; samer.ezziddin@uks.eu; 5 Department of Internal Medicine, University Hospital Ruhr-University Bochum, In der Schornau 23-25, 44892 Bochum, Germany; jan.best@kk-bochum.de; 6 Department of Internal Medicine, University of Duisburg-Essen, Hufelandstr. 55, 45147 Essen, Germany; 7 Department of Diagnostic and Interventional Radiology and Neuroradiology, University of Duisburg-Essen, Hufelandstr. 55, 45147 Essen, Germany; juliane.goebel@uk-essen.de; 8 MVZ CDT Strahleninstitut GmbH, Turiner Straße 2, 50668 Cologne, Germany

**Keywords:** selective internal radionuclide therapy (SIRT), ^90^Y glass microspheres, hepatocellular carcinoma (HCC), transarterial chemoembolization (TACE)

## Abstract

Transarterial chemoembolization (TACE) is currently the standard of care in patients with unresectable hepatocellular carcinoma (HCC), and selective internal radionuclide therapy (SIRT) with ^90^Y microspheres is mainly used as an alternative modality in patients considered poor candidates for TACE. Treatment with sorafenib is the recommended option for patients with progressive disease after TACE. This study aims to evaluate the safety and efficacy of SIRT with glass microspheres in patients with progressive HCC after repeated TACE who are not eligible for treatment with sorafenib. Forty-seven patients with progressive HCC after a median of three TACE sessions (range 2–14) underwent SIRT (3.5 ± 1.5 GBq; liver target dose 110–120 Gy). Toxicity was recorded 4 and 12 weeks after treatment and reported according to the Common Terminology Criteria for Adverse Events Version 5.0. Treatment response was assessed three months after SIRT using multiphase computed tomography and modified criteria in solid tumors (mRECIST). Survival analyses were performed using Kaplan–Meier curves and a Cox proportional hazards model for uni- and multivariate analyses. Significant but reversible hepatotoxicity (≥grade 3) occurred in five patients (11%). No radioembolization-induced liver disease (REILD) was observed. The number of previous TACE sessions and cumulative administered activity did not predict the incidence of post-SIRT significant hepatotoxicity. Treatment responses consisted of partial responses in 26 (55%), stable disease in 12 (26%), and progressive disease in 9 (19%) patients. The median overall survival (OS) was 11 months (95% confidence interval (CI), 9–13), and objective responses to SIRT were associated with a longer OS (*p* = 0.008). Significant hepatotoxicity (≥grade 3) after SIRT was a contributor to impaired survival (median OS 6 months (95% CI, 4–8) vs. 12 months (95% CI, 10–14), *p* < 0.001). SIRT with glass microspheres is a safe and effective salvage treatment for patients with progressive HCC refractory to TACE who are considered poor candidates for sorafenib treatment.

## 1. Introduction

The current Practice Guidance by the American Association for the Study of Liver Diseases recommends further diagnostic work-up for hepatocellular carcinoma (HCC) in the presence of a hepatic lesion >1 cm and an increase in alpha-fetoprotein >20 ng/mL in serum. Multiphase CT or MRI are used as imaging modalities, and histological analysis represents the gold standard [1]. In unresectable HCC, palliative liver-directed treatment options like transarterial chemoembolization (TACE) or selective internal radionuclide therapy (SIRT) can significantly reduce hepatic tumor burden and may increase survival in patients with liver-dominant disease [2,3,4].

TACE is currently the standard treatment for patients with locally advanced HCC without vascular invasion or extrahepatic spread (intermediate stage) [3,5,6,7]. However, a sufficient response after a single TACE session is rare, and often repeated TACE is required to achieve a good response [8]. Accordingly, at least two TACE sessions should be performed before abandoning the procedure [9]. Despite increasing evidence supporting the favorable efficacy of SIRT with ^90^Yttrium (^90^Y) microspheres in patients with intermediate to advanced HCC [10,11,12,13,14,15], the lack of prospective randomized clinical trials has currently limited its role as an alternative method for patients considered poor candidates for TACE. For patients refractory to repeated TACE, systemic treatment with the multikinase inhibitor sorafenib is recommended, but sometimes with suboptimal tolerability outweighing the survival benefits [16,17,18]. This leaves SIRT as the only treatment option after failure of TACE in this setting [19,20].

Repeated TACE can be associated with vascular injury and sometimes with liver function deterioration. Furthermore, profound TACE-induced tumor dearterialization may reduce the selective deployment of ^90^Y microspheres inside the tumor vasculature [21,22,23]. Therefore, SIRT might be associated with a higher risk of treatment failure and severe hepatic toxicity in patients previously treated with repeated TACE. Conversely, a progressive tumor probably develops new tumor vessels, which might compensate for the TACE-induced devascularization. Thus, this study aims to assess the safety and efficacy of SIRT with glass ^90^Y microspheres (TheraSphere™, Boston Scientific Corporation, Ottawa, ON, Canada) in patients with progressive HCC refractory to repeated TACE but not eligible for sorafenib treatment.

## 2. Results

### 2.1. Toxicity

The mean treatment activity per patient was 3.3 ± 1.5 GBq, and the mean follow-up time was 17 ± 2 months. Three of the 47 patients were still alive at the time of analysis. Recorded acute adverse events were as follows: fatigue in 18 (38%), nausea without vomiting in 10 (21%), fever in 9 (19%), and transient abdominal pain in 5 patients (11%). No patient needed hospitalization due to the reported adverse events, and all symptoms resolved within the first six weeks after SIRT. Prior to the treatment, 24 patients had impaired liver function (grade I: 24, grade II: 7, ≥grade III: 0). Post-SIRT hepatotoxicity was defined as newly impaired liver function (albumin, bilirubin, AST/ALT, INR, ascites) or as deterioration in CTCAE-grading after SIRT. Post-SIRT hepatotoxicity was observed in 36 patients. In 10 patients, liver function parameters deteriorated (grade II), and in 5 patients, a significant new hepatic toxicity of grade III–IV occurred. Detailed information about toxicity after treatment is given in Table 1: 12 patients showed elevated liver transaminase (10 grade I, 2 grade II) within six weeks after treatment, and 20 patients had biliary toxicity (4 grade I; 12 grade II and 4 grade III–IV). Fourteen patients showed relevant hepatic toxicity (grade II) based on both liver transaminase and bilirubin concentrations. Portal vein thrombosis and high hepatic tumor load (≥25%) were the independent contributing factors to treatment-induced significant hepatotoxicity, as depicted in Table 2.

**Figure 1:**
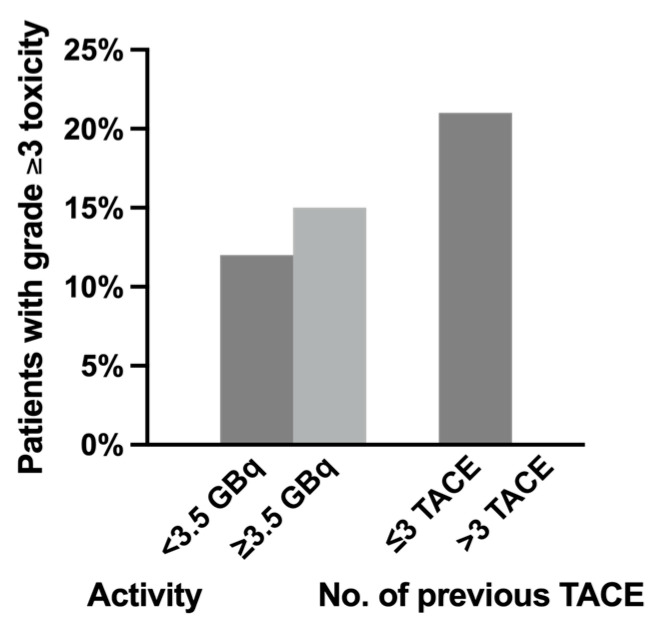
The relation of significant (grade ≥3) hepatotoxicity and (1) administered activity and (2) number of previous transarterial chemoembolization (TACE) sessions.

All five patients with newly induced hepatic toxicity had ≤3 TACE sessions prior to SIRT, and no increase in the incidence of significant toxicity was observed in patients with >3 prior TACE sessions (Figure 1). High cumulative activity (≥3.5 GBq) during SIRT and a higher number of previous TACE sessions was also not associated with increased hepatic toxicity (*p* = 0.706). Significant hepatic toxicity was resolved within 12 weeks in all but one patient who died because of acute renal failure. No severe radioembolization-induced liver disease (REILD) was documented, which was defined as new relevant serum total bilirubin elevation (≥3 mg/dL) combined with new ascites 1–2 months after treatment without tumor progression or bile duct obstruction. No radiation-induced pneumonitis, gastroduodenal ulceration, or other organ toxicity was observed.

### 2.2. Response and Survival

**Figure 2:**
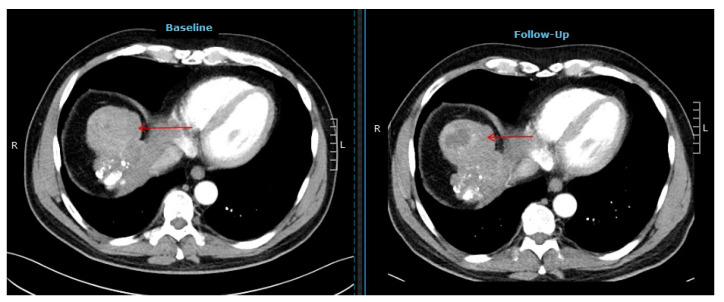
Computer tomography images of a patient showing a partial response after selective internal radiotherapy (red arrow indicates the tumor lesion).

Restaging according to mRECIST yielded a partial remission (PR) in 26 (55%), stable disease (SD) in 12 (26%), and progressive disease (PD) in 9 (19%) patients. Complete remission was not observed in our cohort. An example of a patient with a partial response according to mRECIST is displayed in Figure 2.

**Figure 3:**
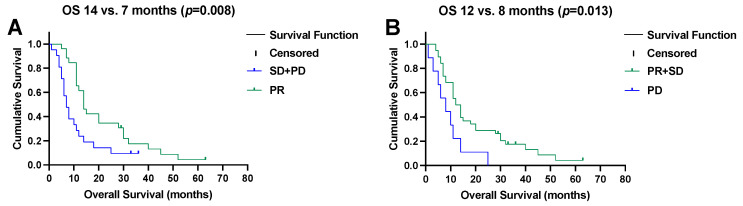
(**A**) Objective remission (partial response (PR)) after selective internal radiotherapy prolongs overall survival (OS), and (**B**) early progressive disease (PD) impairs overall survival.

The median time to progression after SIRT was 7 months (95% CI, 6–8) and the median OS was 11 months (95% CI, 9–13). Patients showing objective responses to SIRT (i.e., PR) had a median OS of 14 months (95% CI, 11–17) as opposed to 7 months (95% CI, 5–9) in the remaining patients (*p* = 0.008), as illustrated in Figure 3A. In contrast, progression after SIRT (i.e., PD) was associated with a shorter survival (median OS, 8 months (95% CI, 2–14) versus 12 months (95% CI, 9–15), *p* = 0.013, Figure 3B).

**Figure 4:**
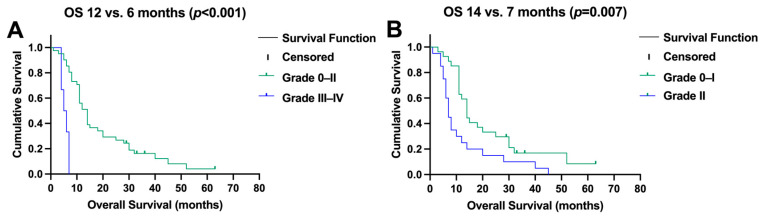
(**A**) Significant (grade ≥ 3) toxicity after selective internal radiotherapy reduced survival, and (**B**) moderate changes in liver function (CTC II) impaired overall survival (OS).

Significant hepatotoxicity (≥grade III) after SIRT was also a contributor to impaired survival (median OS, 6 months (95% CI, 4–8) versus 12 months (95% CI, 10–14), *p* < 0.001, Figure 4A). Patients developing grade II hepatotoxicity showed a trend towards a shorter survival (median OS, 7 months (95% CI, 6–8) versus 14 (95% CI, 11–17), *p* = 0.007, Figure 4B). 

Among the baseline characteristics, a relevant decrease in hepatic functional reserve (≥grade II) was the only independent predictor of survival, as depicted by the multivariate analysis (HR; 95% CI, 5.5 (1.5–19.9), *p* = 0.009). The analysis of various baseline factors for potential contribution to OS is shown in Table 3.

## 3. Discussion

This retrospective study provides the first results regarding the risk factors for the hepatotoxicity of SIRT with glass-based ^90^Y microspheres (TheraSphere^™^) in a well-characterized patient cohort (*n* = 47) with unresectable HCC failing repeated TACE. In clinical practice, previous TACE is considered a major risk factor for serious SIRT-induced toxicity. Fitting to this, the detected rate of significant hepatotoxicity in our cohort (11%) was higher than the previously reported rate of 4–9% in treatment-naive patients or heterogeneous cohorts regarding previous treatment modalities [2,24,25,26,27,28,29,30,31,32,33]. However, treatment-induced hepatotoxicity was almost always reversible, and liver function parameters returned to pre-treatment levels in all but one patient. 

Portal vein thrombosis (PVT) is an absolute contraindication for TACE. On the contrary, SIRT with glass microspheres has a very low embolic tendency and can be safely applied to patients with PVT [34,35,36]. Although PVT was associated with a higher incidence of moderate hepatotoxicity in our cohort, the survival outcome of patients who had developed PVT under TACE did not differ from patients without PVT. Among all other analyzed baseline characteristics, only a hepatic tumor load of >25% was a risk factor for significant hepatotoxicity. This observation is in line with the reported data after treatment with ^90^Y resin microspheres in a large prospective observational study (CIRT study). In a recent study on 1027 patients in a heterogenous patient cohort with various liver tumors, including HCC in 422 patients, a tumor load of >20% was a significant predictor of increased hepatotoxicity (*p* = 0.0283) [37]. Notably, the number of previous TACE sessions was not a predictor of hepatotoxicity in our cohort, encouraging the consideration of SIRT in patients heavily pre-treated with TACE, similar to previous findings in a smaller patient group (*n* = 29), indicating the suitability of SIRT after TACE not to be limited by increased risk of toxicity [38].

Achievement of disease control in 81% is promising and compares favorably with the other treatment modalities. Sorafenib, as the recommended agent for TACE-refractory HCC lesions [16,17,39,40], is commonly associated with adverse effects leading to treatment interruption or even permanent drug discontinuation [16,17,39]. Moreover, an objective radiological response is rarely observed after treatment with sorafenib. The reported overall survival in the main clinical phase III trial of sorafenib (SHARP trial) was 10.7 months [17]. Although comparing our retrospective data with results from prospective trials is of limited validity, the objective response rate (ORR) of 55% and median OS of 11 months (CI 95%, 9–13) in our patients ineligible for treatment with sorafenib is very encouraging. Furthermore, responders (i.e., PR) had a significantly longer survival in our cohort (median OS 14 versus 7 months, *p* = 0.008), underlining the impact of ORR on the survival outcomes of HCC patients after SIRT [15]. The rate of hepatotoxicity in our study was lower than the reported rate after sorafenib treatment. Johnson et al. analyzed the efficacy and safety of SIRT as a salvage therapy after ≥1 TACE, resulting in a slightly inferior OS of 8.4 months; however, BCLC stage C was more prevalent in their cohort (72.5% vs. 47%) [38]. Fitting to this, in a study by Reeves et al., a subgroup of BCLC stage B patients with 1–7 TACE before SIRT (*n* = 7) reached an OS of 14.8 months [41].

In addition to tumor progression, therapy-induced hepatotoxicity may affect the survival of patients with HCC [15,17]. In a retrospective study, grade II toxicity has been suggested as a risk factor for poor survival outcomes. Correspondingly, relevant hepatotoxicity impaired the survival outcome in our cohort (*p* < 0.007). Significant hyperbilirubinemia (grade III/IV), as a hallmark of REILD, has been reported in 14% of patients undergoing ^90^Y glass microsphere SIRT [24]. Although 9% of our cohort developed grade III transient biliary toxicity, no REILD was observed in our cohort.

Our findings support the application of SIRT with glass microspheres after undergoing repeated TACE who are ineligible for treatment with sorafenib. SIRT could induce disease stabilization in most patients, leading to an improved survival outcome. Hepatotoxicity was reversible and the number of previous TACE was not a risk factor. However, the retrospective design and small patient group limit the statistical power and ability to generalize from our results regarding the subgroup analysis and baseline factors with a potential impact on survival. Furthermore, pathological data were, unfortunately, not available to be included in this clinical observational study. It would be interesting to analyze pathological parameters and the treatment efficacy, which might be the subject of further studies.

## 4. Materials and Methods

### 4.1. Patient Characteristics

Forty-seven patients with TACE-refractory HCC (38 men, 9 women; age range: 40–85 years; mean age: 69 years) treated with SIRT in the Department of Nuclear Medicine, University Duisburg-Essen, were included in this retrospective analysis [42]. The decision to perform SIRT was based on interdisciplinary consent after discussion in a multidisciplinary tumor board. All patients had progressive liver tumors despite repeated TACE procedures (median: 3, range 2–14) and were not suitable for sorafenib treatment. Apart from repeated TACE, previous treatments were comprised of radiofrequency ablation (*n* = 9), surgical resection (*n* = 7), liver transplantation (*n* = 4), and transarterial embolization or ethanol injection (*n* = 2). All patients fulfilled the general inclusion criteria for radioembolization [43,44]. Twenty-three patients presented with a unilobar, and 24 patients with a bilobar hepatic tumor spread. At the time of SIRT, 25 patients were classified as stage B and 22 as stage C according to the Barcelona Clinic Liver Cancer (BCLC) staging classification. In 39 patients, HCC was confined to the liver, while 8 patients showed a liver-dominant disease with extrahepatic metastases. In these eight patients, the tumor board identified the extrahepatic metastases as not predominately prognostically relevant regarding survival, size, quantity, and localization of the metastases. The baseline patient characteristics are presented in Table 4. The local committee on ethics approved this retrospective study, and all subjects signed a written informed consent to treatment prior to evaluation and radioembolization session.

### 4.2. Radioembolization Procedure

Intra-abdominal and excessive pulmonary (lung-shunt fraction) deposition were excluded prior to radioembolization by a pre-treatment diagnostic angiogram with planar and SPECT/CT ^99m^Technetium-HSA (human serum albumin microspheres) imaging after an intra-arterial injection of 150 MBq of ^99m^Tc-HSA [45]. Radioembolization was performed within an interval of 1–4 weeks following diagnostic angiography using glass-based ^90^Y microspheres (TheraSphere™). The prescription of activity was derived from the MIRD-based dose calculation method provided by the manufacturer (Boston Scientific Corporation, Marlborough, MA, USA, former BTG plc, London, UK, former Nordion Inc., Ottawa, ON, Canada) to achieve a standard target dose of 100–120 Gy. The liver was treated in a single session (unilobar, *n* = 23, whole liver, *n* = 1) or in a sequential lobar fashion (*n* = 23 patients). Post-treatment ^90^Y bremsstrahlung imaging was performed to document target accumulation. Parameters for liver function (albumin, bilirubin, AST/ALT, INR, ascites) were determined before as well as 4 and 12 weeks after each SIRT. Hepatic toxicity was classified according to the Common Terminology Criteria for Adverse Events Version 5.0 (CTCAE v.5.0). Morphological response to SIRT was assessed using contrast-agent-enhanced computed tomography (CT, early arterial and venous phase) 3 months after SIRT using modified response criteria in solid tumors (mRECIST) [46,47].

### 4.3. Statistical Analysis

Statistical analyses were performed using the SPSS software package version 29.0 (IBM, Armonk, NY, USA). Graph-Pad Prism version 10.1 (GraphPad Software, San Diego, CA, USA) was used to plot graphs. The results were presented as mean ± standard deviation for continuous variables; categorical variables are presented as frequencies with respective percentages. The association of treatment-induced hepatic toxicity (grade I–IV) with the baseline characteristics of the study population, number of previous TACE sessions, and administered activity were examined, applying non-parametric tests for independent samples as well as multiple regression analysis. Survival assessment from the start of radioembolization was performed using the Kaplan–Meier method. Overall survival (OS) was assessed from the first radioembolization session, and the death of patients was considered as an event for OS irrespective of the cause. Survival outcomes were stratified by various variables and compared using the log-rank test. Multivariate analysis (Cox proportional hazards model) was performed with those variables showing at least a trend (*p* < 0.1) of influence on the univariate analysis (log-rank test). A *p* value < 0.05 was considered significant.

## 5. Conclusions

SIRT with glass microspheres is an effective salvage treatment in patients with progressive HCC refractory to TACE who are ineligible for treatment with sorafenib. SIRT provides disease stabilization and improves survival. The rate of significant hepatotoxicity was acceptable, considering the lack of alternative treatment options. Furthermore, the number of previous TACE sessions should not preclude the consideration of SIRT in heavily pre-treated patients fulfilling the established prerequisites.

## Figures and Tables

**Table 1:** Toxicity after SIRT according to the CTCAE v.5.0.

Toxicity (Grade)	Post-SIRT Altered LFT, *n* (%)			SIRT-Induced Toxicity, *n* (%)		
	I	II	III–IV	I	II	III–IV
Bilirubin	9 (19)	14 (30)	4 (9)	4 (9)	12 (26)	4 (9)
Albumin	7 (15)	6 (13)	0 (0)	4 (9)	5 (11)	0 (0)
AST/ALT	33 (70)	4 (9)	0 (0)	12 (26)	2 (4)	0 (0)
AST	30 (70)	4 (9)	0 (0)	10 (21)	2 (4)	0 (0)
ALT	24 (51)	0 (0)	0 (0)	12 (26)	0 (0)	0 (0)
INR	13 (28)	3 (6)	0 (0)	9 (19)	3 (6)	0 (0)
Creatinine	0 (0)	0 (0)	3 (6)	0 (0)	0 (0)	2 (4)
Ascites	8 (17)	3 (6)	0 (0)	3 (6)	3 (6)	0 (0)
Toxicity of any kind	22 (47)	14 (30)	7 (15)	21 (45)	10 (21)	5 (11)

SIRT: selective internal radiation therapy; LFT: liver function test; AST: aspartate aminotransferase; ALT: alanine transaminase; INR: International Normalized Ratio.

**Table 2:** Contributing factors to toxicity after SIRT.

		Statistical Analysis		
		Univariate *p* Value	Multivariate *p* Value (95% CI)	
Age	≤65 years	0.402		
	>65 years			
Tumor load	≤25%	0.041	0.029	0.023–0.398
	>25%			
Cumulative activity	<3.5 GBq	0.706		
	≥3.5 GBq			
Tumor spread	Unilobar	0.227		
	Bilobar			
Hepatitis	No	0.256		
	Yes			
BCLC staging	B	0.307		
	C			
Child classification	A	0.559		
	B			
Lymph node involvement	No	0.224		
	Yes			
Hepatitis	No	0.256		
	Yes			
PVT	No	0.074	0.029	0.024–0.420
	Yes			
Altered LFT	Grade 0–I	0.283		
	Grade II–IV			
Pre-treatment	RFA	0.364		
	Resection/LT			
	Embolization/PEI			

BCLC: Barcelona Clinic Liver Cancer; Child classification: Child–Pugh system; PVT: portal vein thrombosis; LFT: liver function test; RFA: radiofrequency ablation; LT: liver transplantation; PEI: percutaneous ethanol ablation.

**Table 3:** Contributing factors to survival after SIRT.

		Survival Analysis		Statistical Analysis		
		Median OS	HR (95% CI)	Univariate *p* Value	Multivariate HR (95% CI), *p* Value	
Age	≤65 years	14	11–17	0.685		
	>65 years	14	5–23			
Tumor load	≤25%	14	10–18	0.518		
	>25%	14	11–17			
Cumulative activity	<3.5 GBq	14	8–20	0.323		
	≥3.5 GBq	14	13–15			
Tumor spread	Unilobar	14	6–22	0.620		
	Bilobar	14	12–16			
Hepatitis	No	15	10–20	0.247		
	Yes	11	3–19			
BCLC staging	B	13	11–15	0.389		
	C	8	3–14			
Child classification	A	12	10–15	0.736		
	B	10	6–14			
Lymph node involvement	No	11	9–13	0.686		
	Yes	11	0–39			
Hepatitis	No	12	9–15	0.288		
	Yes	10	4–16			
PVT	No	13	11–15	0.352		
	Yes	7	3–11			
Altered LFT	Grade 0–I	12	10–14	0.002	5.5	0.009
	Grade II–IV	6	5–8		(1.5–19.9)	
Toxicity after SIRT	Grade 0–II	14	12–17	<0.001		
	Grade III–IV	6	5–7			

OS: overall survival; HR: hazard ratio; CI: confidence interval; BCLC: Barcelona Clinic Liver Cancer; Child classification: Child–Pugh system; PVT: portal vein thrombosis; LFT: liver function test; SIRT: selective internal radiation therapy.

**Table 4:** Baseline patient characteristics.

	All Patients (*n* = 47)
Age	
>65 years	32 (68)
≤65 years	15 (32)
Hepatic tumor load	
>25%	23 (49)
≤25%	24 (51)
Cumulative applied activity during SIRT session(s)	
≥3.5 GBq	21 (45)
<3.5 GBq	26 (55)
Hepatic tumor spread	
Bilobar	24 (51)
Unilobar	23 (49)
BCLC staging	
Stage C	22 (47)
Stage B	25 (53)
Child classification	
Child B	21 (45)
Child A	26 (55)
Extrahepatic lymph node metastasis	
No	39 (83)
Yes	8 (17)
Hepatitis	
Yes	21 (45)
No	26 (55)
Etiology of hepatitis	
Alcohol-related	6 (13)
NASH	12 (26)
Viral	18 (38)
Cryptogenic	11 (23)
PVT	
Yes	16 (33)
No	31 (66)
Pre-treatment	
RFA	9 (19)
Embolization/PEI	2 (4)
Resection/LT	11 (24)
LFT in all patients	
Total bilirubin (mg/dL, normal range: 0.3–1.0)	1.0 ± 0.5
Albumin (g/dL, normal range: 3.4–5.4)	3.9 ± 0.5
AST (U/L, normal range: 5–40)	77.6 ± 62.4
ALT (U/L, normal range: 7–56)	59.5 ± 41.1
INR (normal range: 0.8–1.1)	1.1 ± 0.1
Altered LFT (CTC I)	
Total bilirubin (>ULN–1.5 × ULN, mg/dL)	16 (34)
Albumin (<LLN–3 g/dL)	6 (13)
AST/ALT (>ULN–3 × ULN, U/L)	26 (55)
INR (>1.2–1.5 × baseline)	4 (9)
Ascites	5 (11)
Altered LFT (CTC II)	
Total bilirubin (>1.5–3.0 × ULN, mg/dL)	2 (4)
Albumin (3–2 g/dL)	2 (4)
AST/ALT (>3–5 × ULN, U/L)	2 (4)
INR (>1.5–2.5)	0 (0)
Ascites	0 (0)

Data presented as *n* (%). SIRT: selective internal radiotherapy; GBq: gigabecquerel; BCLC: Barcelona Clinic Liver Cancer; Child classification: Child–Pugh system; NASH: non-alcoholic steatohepatitis; PVT: portal vein thrombosis; RFA: radiofrequency ablation; PEI: percutaneous ethanol ablation; LT: liver transplantation; LFT: liver function test; CTC: Common Terminology for Common Adverse Events (v.5.0); ULN: upper limit of normal; LLN: lower limit of normal; AST: aspartate aminotransferase; ALT: alanine transaminase; INR: International Normalized Ratio.

## Data Availability

The datasets analyzed and/or analyzed during the current study are available from the corresponding author on reasonable request.
